# Nanoscale plasmonic wires with maximal figure of merits as a superior flexible transparent conducting electrode for RGB colors

**DOI:** 10.1038/s41598-022-14756-z

**Published:** 2022-06-30

**Authors:** Chin-Chien Chung, Dong-Sheng Su, Tsung-Yu Huang, Cheng-Yi Lee, Robert Jan Visser, B. Leo Kwak, Hyunsung Bang, Chung-Chia Chen, Wan-Yu Lin, Ta-Jen Yen

**Affiliations:** 1 Department of Materials Science and Engineering, National Tsing Hua University, Hsinchu, 30013 Taiwan, ROC; 2 Department of Materials Engineering, Ming Chi University of Technology, New Taipei, 24301 Taiwan, ROC; 3 Advanced Technology Group, Corporate CTO Office, Applied Materials, Santa Clara, CA USA

**Keywords:** Nanowires, Nanophotonics and plasmonics

## Abstract

Based on incredibly increasing applications in modern optoelectronic devices, the demand for securing a superior conductive transparent electrode (TCE) candidate becomes significant and urgent. However, boosting both transmittance and conductance simultaneously is an intrinsic limitation. In this work, we present silver nanoscale plasmonic wires (Ag NPWs) to function as TCEs in the visible light region by lowering their corresponding plasma frequencies. By carefully designing geometric dimensions of the Ag NPWs, we also optimize the performance for red, green, and blue colors, respectively. The demonstrated figure of merits for RGB colors appeared respectively 443.29, 459.46, and 133.78 in simulation and 302.75, 344.11, and 348.02 in experiments. Evidently, our Ag NPWs offer much greater FoMs beyond conventional TCEs that are most frequently comprised of indium tin oxide and show further advantages of flexibility and less Moire effect for the applications of flexible and high-resolution optoelectronic devices.

## Introduction

Currently, transparent conducting electrodes (TCEs) are omnipresent in our daily lives, mainly due to their pivotal applications in solar cells (SCs)^1–6^, light-emitting diodes (LEDs)^3,7–15^, touch panels^13,16,17^, and others. TCEs, namely, should exhibit great optical transparency and electrical conductivity simultaneously, but these two fundamental physical properties intrinsically contradict each other. The reason for such a dilemma is that free electrons in materials not only conduct electricity but also screen incident waves. Although rare, researchers found that some ceramics such as indium tin oxides (ITOs) conduct electricity while maintaining optical transparency because of their oxygen defects inside. Thus, ITO prevails in various types of optoelectronic devices because it promises concurrent 85% transmittance at visible light regime and a sheet resistance lower than 100 Ω/sq^18^. Nevertheless, ITO suffers from several inherent limitations, including material scarcity, toxicity, frangibility^17,19–21^, low output efficiency due to its higher refractive index^22,23^, and high-temperature fabrication procedure. These limitations became a trigger for researchers to seek alternatives, especially for those who could meet the demands for the next generation of flexible and high-resolution display panels^24–26^.

Until now, industries and academia have been investing much effort in developing an alternative to replace ITOs, including carbon nanotubes (CNTs)^8,19,20,27–29^, graphene^11,19,30,31^, and metal wires^2,32–38^. These alternatives show their own advantages, for example, high mechanical strengths and flexibility with abundant material resources for randomly oriented networks of CNTs^8,20^, decent sheet resistance of 100–1000 Ω/sq and 80% transmittance at visible regime^29,30^ for single-layered graphene, and high conductivity and ductility for the metallic wires fabricated by either bottom-up electrospinning^32,33^ or top-down lithographic^34,35,38^ processes. Unfortunately, there still appear several insufficiencies among these methods. First, CNT thin films show lower transmittance and higher sheet resistance when compared with ITOs^4^. Second, the sheet resistance of single-layered graphene remains too high for practical applications of photovoltaic and optoelectronic devices; so, some researchers proposed to enhance their conductivity through multilayered graphene. However, adding a graphene layer would inevitably reduce 3% transmittance. Also, many grain boundaries and dislocations were developed under a large area fabrication process, thus further lowering their conductivity accordingly. Hence, metallic wires are the most promising solution for the next-generation TCEs.

The reported state-of-the-art micron-scale metallic structures achieved 90% transmittance and 10 Ω/sq sheet resistance for the electrospinning process^39^, and 88.6% transmittance and 2.1 Ω/sq^40^ sheet resistance for the lithographic procedure. These two metal wires have been then integrated into solar cell^41^ and OLED^42^ applications. Nevertheless, once the pixel size of OLED displays approaches submicron scale, these two techniques appear critical insufficiencies. For example, randomly electrospinning wires with multiple junctions lead to fluctuation of transmittance and sheet resistance; furthermore, their random distribution makes them not suitable in the application of high-resolution arrayed OLEDs. Conversely, microscale metallic meshes suffer from Moiré fringes, hindering their applications in the field of high-resolution optoelectronic devices including augmented reality and virtual reality. To solve the abovementioned issues, in this work, we proposed two-dimensional plasmonic wires with nanoscale periodicity, termed as nanoscale plasmonic wires (NPWs). Note that although the periodicity of the NPWs approaches the diffraction limit, we can still achieve great transmittance and conductivity simultaneously. We optimized the design of the proposed NPWs, further maximizing their figure of merits (FoMs), presenting a superior TCE for high-resolution OLED displays^43^.

### Design, simulation, and characterization

**Figure 1:**
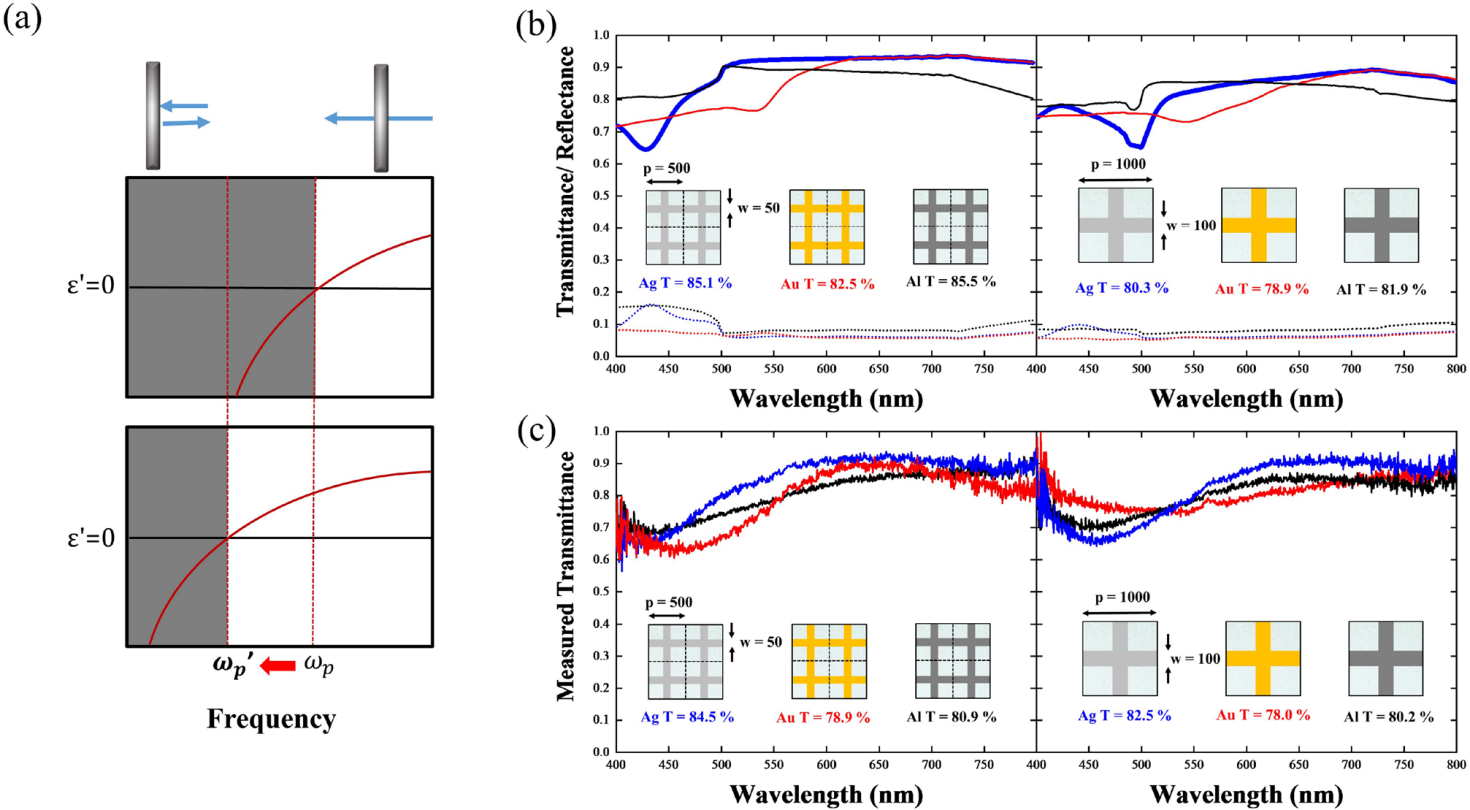
(**a**) Scheme of the mechanism of our proposed nanoscale plasmonic wires (NPWs). By lowering the effective plasmon frequency of the NPWs, we could achieve high optical transparency and good conductivity beyond the plasmon frequency and at direct current, respectively. (**b**) Simulated transmittance (solid line) and reflectance (dashed line) spectra of the NPWs with the periodicity of 500 nm (left panel) and micron-scale metallic meshes with the periodicity of 1000 nm (right panel) for the materials of silver (blue), gold (red), and aluminum (black). (**c**) Measured transmittance spectra of the NPWs (left panel) and the micron-scale metallic meshes (right panel). Note that the packing density of the two is the same for a fair comparison. Here the performance of different metals was examined. Overall, silver outweighs the other two metals in both simulated and measured results.

To reach higher transparency of metallic meshes^41,44^, a straightforward solution is to follow Bethe’s diffraction theory of broadening the size of their openings, particularly much larger than the incident wavelengths^45^. Nonetheless, in this case, the packing density of metallic meshes becomes less, such that the corresponding conductivity certainly becomes too low to be a good TCE. Contrary to broadening the size of the openings, in this study, we designed a metallic structure called plasmonic wires, in which the size of their openings is as small as nanoscale, but we can still achieve excellent optical transparency and electrical conductivity simultaneously. The rationale of these NPWs can be interpreted by combining the Drude model and the effective media theory together^46^ as shown in Eq. (1) below:

1 $$ {\upomega }_{{\text{p}}}^{2} = \frac{{{\text{n}}_{{{\text{eff}}}} {\text{e}}^{2} }}{{{\upvarepsilon }_{0} {\text{m}}_{{{\text{eff}}}} }} = { }\frac{{2{\pi c}_{0}^{2} }}{{{\text{p}}^{2} {\text{ln}}\left( {{\text{p}}/{\text{r}}} \right)}}, $$

where $\omega_{p}$ denotes the plasma frequency of the materials, *n*_*eff*_ the effective density of electrons, *m*_*eff*_ the effective mass of electrons, *p* and *r* the periodicity and the radius of the wires, respectively. Note that $\omega_{p}$ of bulk metals appears beyond visible regime, such that all metals are conductive but not transparent. By replacing bulk metals with the NPWs, one can not only decrease the *n*_*eff*_ because of less packing density but also increase *m*_*eff*_ stemming from mutual inductance among metallic wires. Consequently, we can greatly suppress $\omega_{p}$ to be lower than visible regime, by tailoring the structural parameters of *p* and *r* accordingly, transforming metals to be conductive and transparent simultaneously, as depicted in Fig. 1a.

Not only the structural parameters but also the material properties can influence the performance of the NPWs. Here, we adopted three commonly used metals of silver (Ag), gold (Au), and aluminum (Al), in which the plasmon and damping frequencies for these three materials are 3700/144.7 (Al), 2321/5.513 (Ag), and 2068/4.449 THz (Au)^47^, respectively (see Method for detailed setup in simulation), to construct the NPW-based TCEs (*p* = 500 nm, w = 50 nm, and t = 50 nm) as well as micron-scale metal meshes (*p* = 1000 nm, w = 100 nm, and t = 50 nm) as the controlled groups. Figure 1b shows the simulated transmittances of the NPWs and the controlled metal meshes. Note that for a fair comparison, we fix the packing density of 19% for both cases of the NPWs and micron-scale metal meshes. The periodicity and linewidth for NPWs and micron-scale metal meshes are 500 nm/50 nm and 1 µm/ 100 nm. Average transmittances through the entire visible regime (i.e., 400–800 nm) for Ag-, Au-, and Al-based NPWs and micron-scale meshes are 85.1%, 82.5%, and 85.5%, and 80.3%, 78.9%, and 81.9%, respectively. The transmittances of the NPWs are all greater than the micron-scale metal meshes because we employed effective medium theory to explain the transmissive mechanism of the NPWs. Since the effective permittivity of the NPWs is positive, indicating light could penetrate through the entire area of the NPWs. In contrast, the light penetrates the micron-scale metal meshes through the opening area; thus, only a fraction of the area could contribute the transmittance. Consequently, all the average transmittances of NPW designed by the proposed effective medium theory are greater than those of the micron-scale meshes at the same packing density.

**Figure 2:**
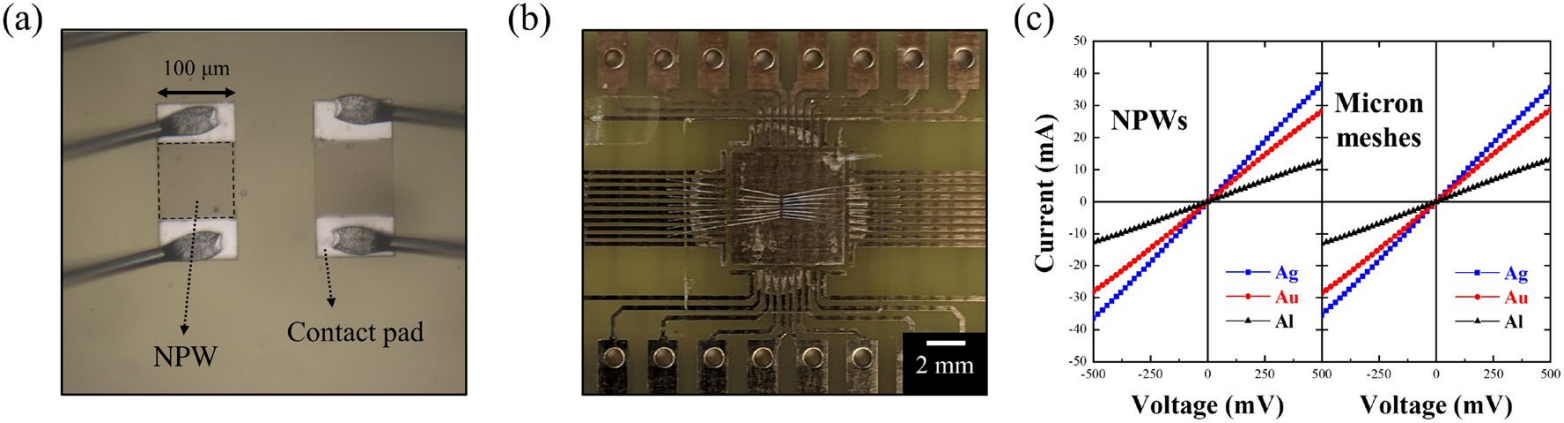
(**a**) Sample wire-bonded to PCB board. (**b**) Optical microscopy image of wire bonding and current–voltage curves for the three different metals of both NPWs and micron-scale meshes.

To validate the performances of our proposed NPWs in experiments and compare them to the simulated results, we then conducted a standard electron beam lithography process with an area of 100 µm × 100 µm for the NPWs with three metals (see Method for the detailed fabrication process). Additionally, to characterize the fabricated sample, we measured the visible light transmittance of the NPWs by a homemade optical measurement setup equipped with a microscope. The measured average transmittance through the entire visible regime for Ag-, Au-, and Al-based NPWs and micron-scale meshes are 84.5%, 78.9%, and 80.9%, and 82.4%, 78.0%, and 80.2%, respectively. The measured transmittance corroborated the prediction from the simulations that indeed the Ag NPWs outperformed the other two metallic NPWs and that the transmittances of the NPWs are all greater than those of the micron-scale metal meshes. Moreover, to examine the NPWs’ performances as TCEs, not only the optical property but also their electric properties matter. To evaluate their electrical properties, we calculated the theoretical resistance of the NPWs by applying Kirchhoff’s rule. For a network with n × n nanowires, its sheet resistance is governed by Eq. (2) listed below:

2 $$ {\text{R}}_{{\text{s}}} = {\uprho }\frac{{\text{n}}}{{{\text{n}} + 1}}\frac{{\text{p}}}{{{\text{wt}}}}, $$

where R_s_ denotes sheet resistance of the NPWs, ${\uprho }$ the resistivity, n the number of metal wires, *p* the periodicity of NPWs, w the metal linewidth, and t the metal thickness, respectively. Through this formula, the NPWs generally provided similar sheet resistance to the micron-scale ones once the packing density is the same. Moreover, the sheet resistances of the NPWs and micron-scale metallic meshes were measured using a multifunctional electrical measurement system (Keithley 2400). Two external pads with a 50 × 100 µm^2^ sandwiching the plural Ag-NPWs area were patterned for the probe contact as depicted in Fig. 2a. Then, the sample was wire-bonded to a printed circuit board for the measurement as shown in Fig. 2b. We collected the current while scanning the voltage in a range of − 500 to 500 mV and then obtained the I-V, the sheet resistance was then calculated by the reciprocal of the slope in Fig. 2b. In measurement, the NPWs revealed a sheet resistance of 13.69, 17.73, and 39.21 Ω/sq as shown in the left panel of Fig. 2c, whereas the micron-scale metal meshes owned a sheet resistance of 14.12, 17.42, and 37.88 Ω/sq as depicted in the right panel of Fig. 2c, which are according to the theoretical calculation.

Finally, to quantitatively evaluate the NPWs in both terms of the optical and electrical properties, one can determine the performances^48^ of the proposed TCEs in the following:

3 $$ {\text{T}} = \left( {1 + \frac{188.5}{{{\text{R}}_{{\text{s}}} }}\frac{{{\upsigma }_{{{\text{opt}}}} }}{{{\upsigma }_{{{\text{dc}}}} }}} \right)^{ - 2} ,{ } $$

where T indicates transmittance of the NPWs, R_s_ the sheet resistance, and σ_opt_ and σ_dc_ the conductivity under optical frequency and direct current conditions, respectively. According to Eq. (3), one can achieve greater transmittance under a specific sheet resistance with smaller $\frac{{\sigma_{opt} }}{{\sigma_{dc} }}$; namely, the performance of TCEs can be regulated by the ratio of $\frac{{\sigma_{dc} }}{{\sigma_{opt} }}$, also known as the FoMs. Table 1 lists the FoMs from the measured results. This table shows that the Ag NPWs outweighed the other two NPWs and the Ag meshes as well. Moreover, because of its better conductivity and better experimental transmittance, silver promises the best NPW-based TCE among the three commonly used metals. Consequently, we would like to focus on the discussion of Ag NPWs for further optimization for RGB colors in organic light-emitting diodes.

**Table 1:** Measured transmittance, sheet resistance, and FoMs of the nano/micron plasmonic wires.

	Ag (nano)	Ag (micron)	Au (nano)	Au (micron)	Al (nano)	Al (micron)
T (%)/R_s_ (Ω/sq)	84.5/13.69	82.4/14.12	79.0/17.73	78.8/17.42	80.9/39.21	80.2/37.88
FoMs	156.22	131.82	85.09	86.01	42.87	42.59

Note that another advantage of using NPWs over micron-scale metal meshes is their ability to suppress the Moire fringes for display applications. Moire fringes appear as a large-scale interference pattern, stemming from overlapping two periodic structures. For example, the periodicity of the overlapped fringes can be described below^49^:

4 $$ {\text{P}}_{{\text{m}}} = \frac{{{\text{P}}_{2} }}{{\sqrt {1 - 2\frac{{{\text{P}}_{2} }}{{{\text{P}}_{1} }}{\text{cos}}\upalpha  + \left( {\frac{{{\text{P}}_{2} }}{{{\text{P}}_{1} }}} \right)^{2} } }}, $$

where P_m_, P_1,_ and P_2_ denote the periodicities of the Moire fringes, display pixels, and TCEs, respectively, and α is the rotation angle between the display and TCEs. Here, we set P_1_ = 1.2 µm for a high-resolution OLED display^43^, P_2_ = 500 nm for NPW, and P_2_ = 1 µm for micron-scale meshes. According to Eq. (4), P_m_ for the NPW indicates 857 nm that is imperceivable for human eyes, but P_m_ appears a noticeable size of 6 µm for micron-scale meshes.

### Optimization for RGB colors

**Figure 3:**
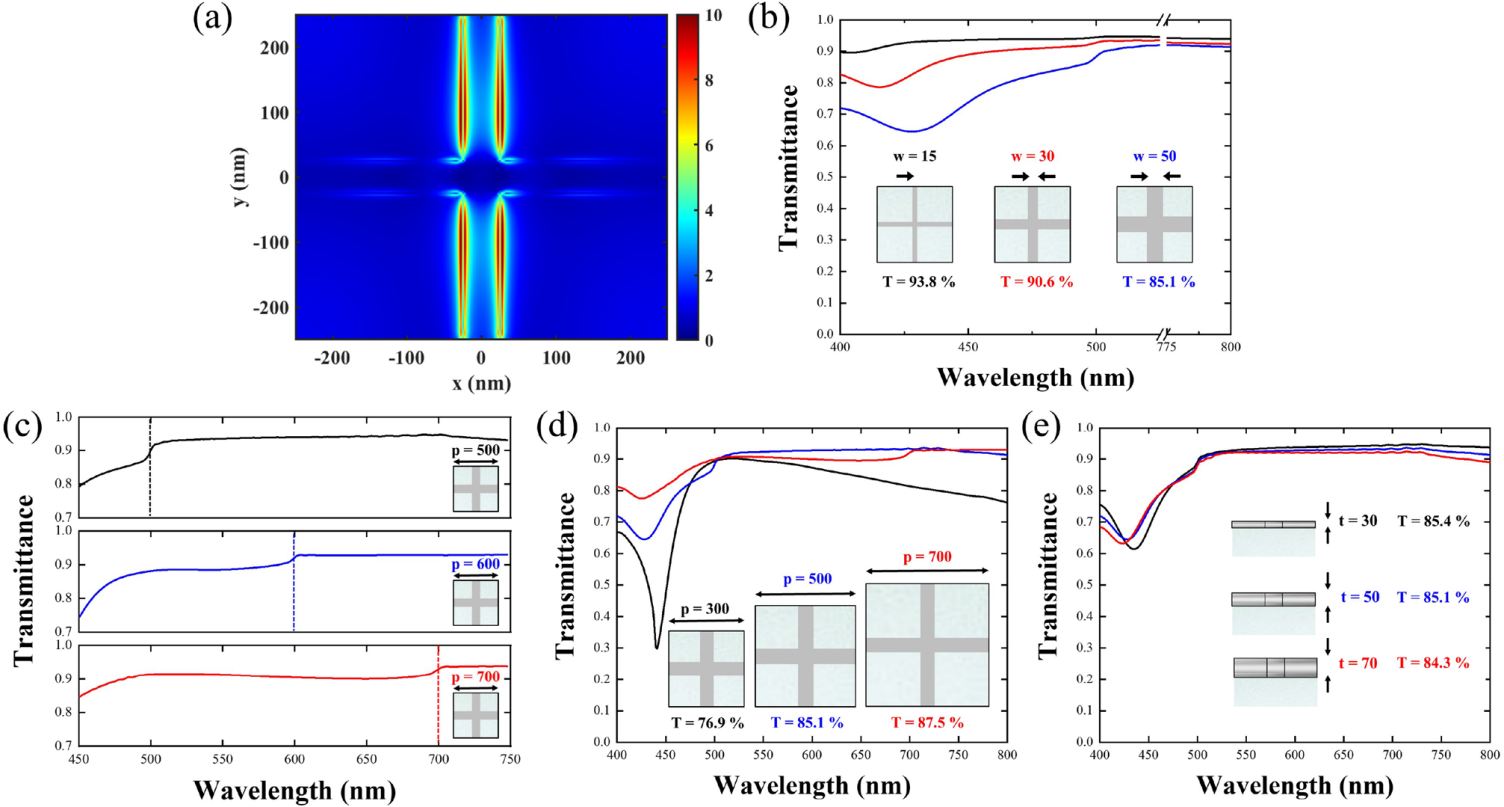
(**a**) Electric field distribution of the Ag NPWs at the wavelength of 430 nm. The field is mainly concentrated on the side-wall of the metallic wires, suggesting excitation of localized surface plasmon along the width of the wires. (**b**) Transmittance spectrum of the Ag NPWs with wire widths of 15, 30, and 50 nm. The dips shifted to a lower wavelength when the width shrinks. (**c**) Transmittance spectra of the silver plasmonic wires with periodicities of 500, 600, and 700 nm, respectively. Diffraction occurs at wavelengths of approximately 500, 600, and 700 nm. (**d**) Transmittance spectra with periodicities of 300, 500, and 700 nm, suggesting that by manipulating the periodicity, we could remove the diffraction from the spectrum. (**e**) Transmittance spectrum of the Ag NPWs with a metal thickness of 30, 50, and 70 nm. Thinner metals reveal higher transmittance.

Overall, the Ag NPWs show good transmittance and sheet resistance compared with the ones of other metals; however, there appear two distinct transmittance dips at the wavelengths of 430 and 500 nm, respectively, as shown in Fig. 1. Thus, we wanted to dig out the origins of these dips and further optimized the behaviors of the Ag NPWs. The first transmittance dip at 430 nm stems from the effect of localized surface plasmon resonance (LSPR). We monitored the field distributions of the Ag-NPW to corroborate the excitation of LSPR. As shown in Fig. 3a, the electric field is mainly concentrated on the sidebars of the Ag-NPW; thus, the excitation frequency of LSPR should be mainly determined by the width of the wire. We then conduct the simulation with three different line widths, i.e., 15, 30, and 50 nm to construct the relationship between the widths and the resonance frequencies. Figure 3b suggests that indeed the narrower width would guarantee the lower resonance wavelength of LSPR; the resonance behavior of LSPR is almost removed from the visible regime for the Ag-NPW with a 15 nm width with a much weaker dip approximately located at approximately 400 nm.

Next, the second transmittance dip at 500 nm is related to the first-order diffraction. We observed the transmittance spectra concerning three different periodicities of 500, 600, and 700 nm, as illustrated in Fig. 3c. In this observation, the wavelength of the dips coincides with the periodicity, evidencing that the cause of the second transmittance dip indeed, results from the first-order diffraction. Such a cause can be further verified by the field profile presented in Fig. S1 in the supporting information. Consequently, one can ease these transmittance dips by deliberately designing the periodicity of the NPWs. For instance, we shrunk the periodicity to be 300 nm for blue-shifting those dips away from visible regions. As shown in Fig. 3d for the NPWs with a periodicity of 300 nm, the transmittance dip caused by diffraction can be removed. Note that the closer the distance between wires, the stronger the LSPR in the blue light regime, which deteriorates the corresponding transmittance. Conversely, with the decreasing periodicity of the NPWs, at the longer wavelength (longer than 700 nm), it also tarnishes the transmittance because of the cutoff of the transverse electric guided mode when the electric field is parallel to the metal lines^21^ (i.e., approximately 500 nm for the periodicities of 300 and 50 nm linewidths). Noteworthily, although at both the longer and shorter wavelengths (i.e., approximately red and blue regimes), the transmittance is poor for the Ag NPWs with a smaller periodicity, the transmittance at the green regime is almost the same; in such a case, the Ag NPWs with a smaller periodicity could provide much better conductivity and greater FoM. We thus gain more freedom to design the NPWs in a specific regime. Finally, Fig. 3e illustrates the transmittance dependence on different metal thicknesses of silver. All transmittance showed similar trends with decreasing average magnitudes while the thickness is increased.

After knowing the origins of the two transmittance dips, here, we wanted to proceed to maximize the FoMs for display applications, at three demanded wavelength ranges of red light (R; 600–700 nm), green light (G; 500–600 nm), and blue light (B; 400–500 nm) regimes. Based on the aforementioned discussions regarding the geometric factors of the width, the periodicity, and the thickness of the Ag NPWs, we tabulated the corresponding sheet resistance and transmittance/FoMs for each wavelength region in Table 2. Note that ITOs with thicknesses of 35 and 70 nm are also included for comparison because 35-nm-thick ITOs are common in thin-film transistors and 70-nm-thick ITOs are with the same thickness as our Ag NPWs. Table 2 presents that the FoMs of the Ag NPWs overwhelmed ITOs, through the entire visible region with the optimized structural parameter of 450/50/70 nm in periodicity, linewidth, and thickness for the red regime and of 400/50/70 nm for both green and blue regimes.

**Table 2:** Simulated transmittance, sheet resistance, and FoMs of the optimized TCEs within three different wavelength ranges.

	R (600–700 nm)	G (500–600 nm)	B (400–500 nm)
Periodicity/linewidth/thickness	450/50/70 nm	400/50/70 nm	400/50/70 nm
T (%)/R_s_ (Ω/sq)	91.7/9.64	91.0/8.57	73.3/8.57
FoMs	443.29	459.46	133.78
FoMs of 35 nm ITO	151.16	111.67	76.34
FoMs of 70 nm ITO	102.04	89.45	87.66

**Figure 4:**
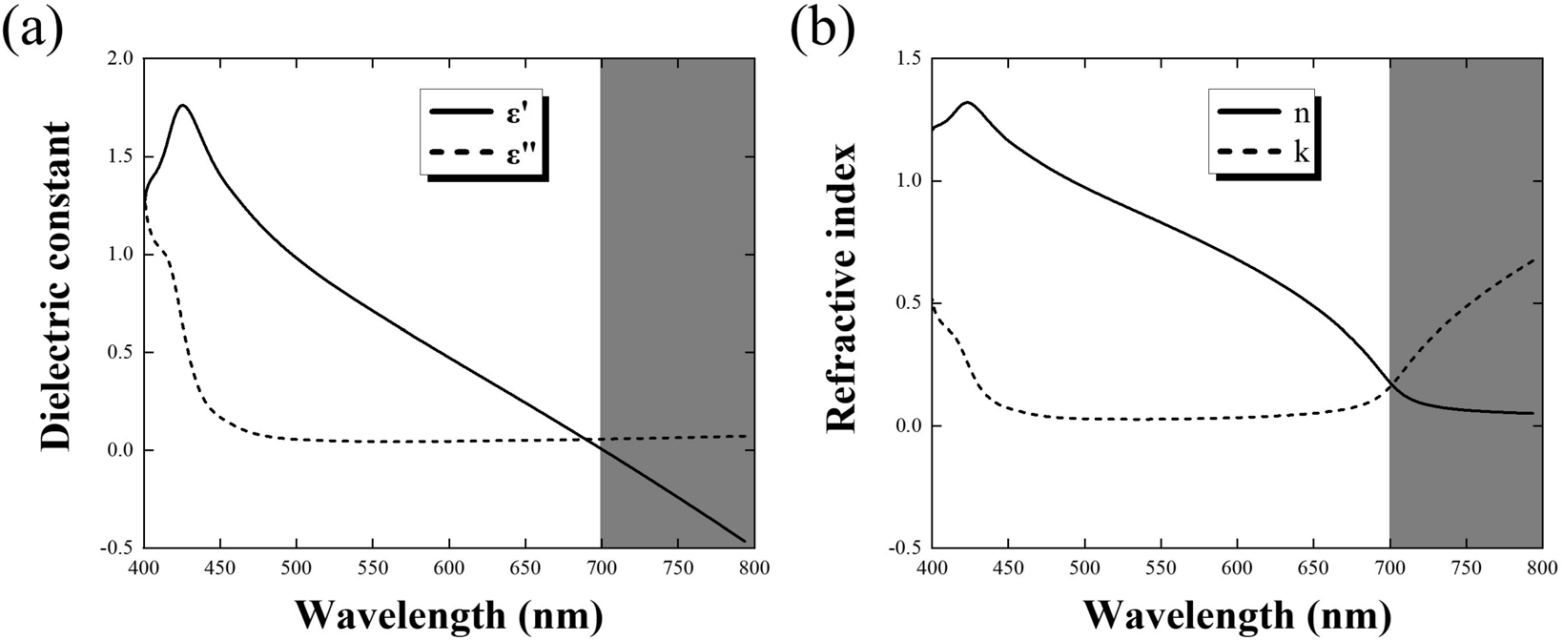
Retrieved (**a**) permittivity and (**b**) refractive index of the proposed Ag NPWs. The effective plasmon frequency is lower down to at a wavelength of 700 nm, confirming our proposed mechanism. Also, the effective refractive index within this frequency range is much lower than the one of ITOs, thus improving the output efficiency of optical devices.

Finally, on the basis of the simulated transmittance and reflectance, we can retrieve the effective permittivity and refractive index of the fabricated Ag NPWs by applying the material property retrieval method^50^. As shown in Fig. 4a, the electric permittivity of Ag should be intrinsically negative in the visible regime because the plasma frequency of Ag appears in the UV regime (i.e., 129 nm). Nevertheless, we found that both the effective permittivity and refractive index of Ag-NPW transform into positive values once the wavelength is longer than 700 nm, i.e., the effective plasmon wavelength of the Ag NPWs was downshifted to 700 nm. Such retrieval results further unveiled that the working principle of our demonstrated Ag NPWs is not the regular transmittance through the wide opening in conventional metal meshes but the down-tuned plasma frequency stemming from decreasing the density of electrons and increasing the effective mass of electrons, as interpreted by the effective medium theory in Eq. (1). In addition, as shown in Fig. 4b, the effective refractive index within this frequency range is much lower than that of ITOs, which leads to better impedance matching with the air and thereby improves the output efficiency of the fabricated Ag NPWs.

**Figure 5:**
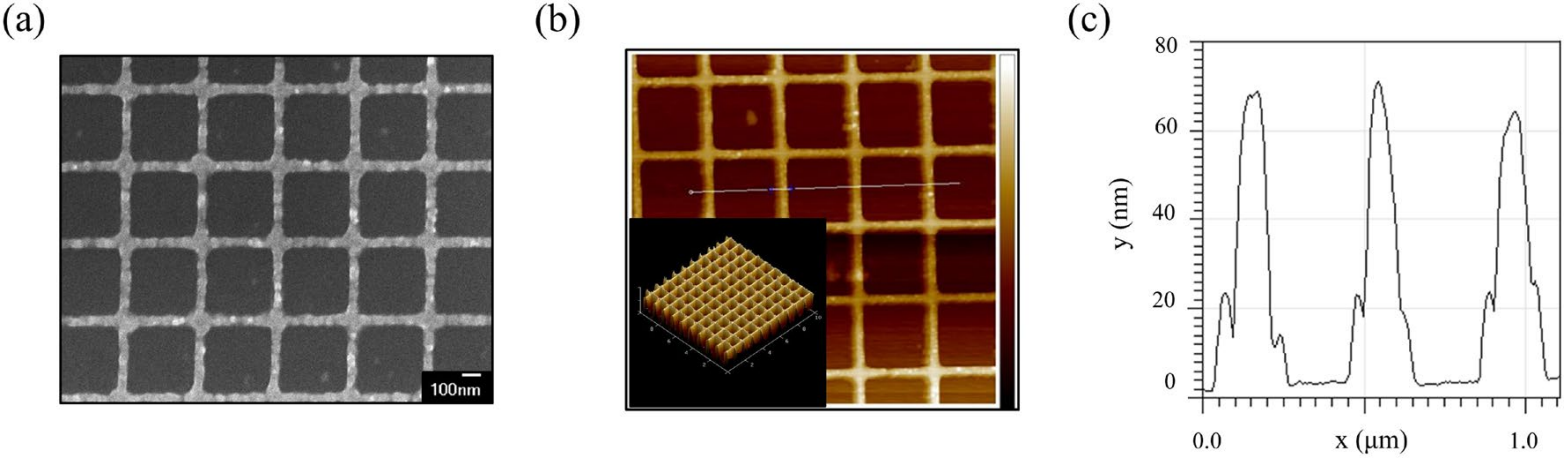
Morphology observation of the fabricated Ag NPWs. (**a**) An SEM image of the fabricated Ag-NPW with parameters of *p* = 400 nm, w = 50 nm, and t = 70 nm, respectively. (**b**) AFM topography of the Ag-NPW. The average thickness is 71.3 nm with good homogeneity. (**c**) Depth profiles along the line labeled in (**b**).

**Figure 6:**
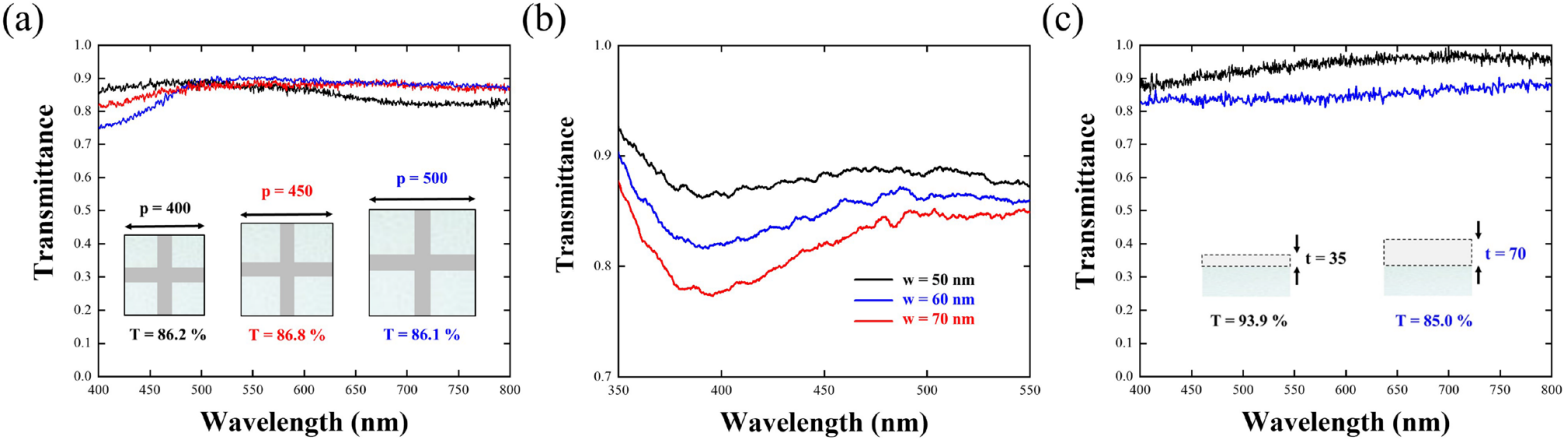
(**a**) Measured transmittance of Ag NPWs with periodicities of 400, 450, and 500 nm and fixed linewidth of 50 nm and thickness of 70 nm. (**b**) Increasing the linewidth (i.e., from 50 to 70 nm) of NPWs with *p* = 400 results in a red-shift of LSPR, experimentally corroborating the simulated results. (**c**) Measured transmittance of 35- and 70-nm-thick ITOs.

Following the promising optimized results from the Ag NPWs, we have learned that the designs of the Ag-NPW TCE with the best FoMs are p/w/t = 400/50/70 nm for both blue and green colors, and 450/50/70 nm for red color, respectively. We thus fabricated these TCEs accordingly and, meanwhile, a TCE with the dimensional parameters of 500/50/70 nm as a comparison to examine the effect of removing first-order diffraction. Figure 5a shows the morphology observation of the fabricated Ag NPWs with the p/w/t of 400, 50, and 70 nm. From the SEM image, the fabricated metal wires are all well-connected to each other without any breaks around joints, which secure a better sheet resistance. Moreover, the topography and line profile of the samples measured via atomic force microscopy was included in Fig. 5b,c, indicating excellent homogeneity of the as-fabricated sample with an average thickness of 71.3 nm. As shown in Fig. 6a, all the measurement results show excellent averaged transmittance of 86.2%, 86.8%, and 86.1%. A minor deviation of slightly lower transmittance was due to rough metal surface and blue-shifted LSPR mode. The measured average transmittance for different periodicities indicates that removing the first-order diffraction greatly enhanced the transmittance in the blue light region. We can find out that Ag NPWs with a periodicity of 400 nm deliver the best blue light transmittance with 88.0%, whereas the ones with a periodicity of 500 nm only deliver blue light transmittance with 81.6%. As for the regime at a longer wavelength, the cutoff of the guided mode suppresses the transmittance of the Ag-NPW with a periodicity of 400 nm as previously discussed, thus contributing to the lowest transmittance (84.3%) at the red light regime. Taking a step further, we also fabricated the Ag NPWs with different linewidths for the periodicity of 400 nm as shown in Fig. 6b. From the transmittance spectra of the NPWs, the LSPR modes are obviously observed and blue-shifted with respect to the decreasing linewidth, experimentally evidencing that by manipulating the linewidth of the NPWs, we could remove the LSPR modes from the visible regime, thus enhancing the average transmittance. Finally, the transmittance of the 35- and 70-nm-thick ITOs is also measured. As depicted in Fig. 6c, the larger thickness of the ITOs results in lower transmittance. Conversely, the corresponding sheet resistances of these three Ag NPWs exhibit 8.18, 9.77, and 10.83 Ω/sq, respectively. Based on the two measurement results, we tabulated the corresponding transmittance and sheet resistance in Table 3 and calculated FoMs in Table 4 for each color. In each light regime, the parameters with the best FoM are according to the optimized simulation result. Although the FoMs are lower than the simulation result that might be due to the deteriorated transmittance, still, the FoMs of our proposed Ag NPWs are much higher than those of ITOs. After all, we summarized the performances of the related works and tabulated them in Table 5.

**Table 3:** Measured transmittance of the optimized TCEs within three different wavelength ranges and sheet resistance.

	R (600–700 nm) (%)	G (500–600 nm) (%)	B (400–500 nm) (%)	(400–800 nm) (%)	${\text{R}}_{{\text{s}}}$ (Ω/sq)
400/50/70 nm	84.3	87.8	88.0	86.2	8.18
450/50/70 nm	88.3	88.0	84.8	86.8	9.77
500/50/70 nm	89.6	88.9	81.6	86.1	10.83
35 nm ITO	92.9	94.7	89.9	93.9	45.03
70 nm ITO	85.5	83.7	83.4	85.0	22.67

**Table 4:** Calculated FoMs from the measured transmittance within three different wavelength ranges and sheet resistance.

	R (600–700 nm)	G (500–600 nm)	B (400–500 nm)	(400–800 nm)
400/50/70 nm	258.18	**344.11**	**348.02**	299.73
450/50/70 nm	**302.75**	292.03	224.05	262.14
500/50/70 nm	286.94	307.89	162.36	223.39
35 nm ITO	151.16	111.67	76.34	132.80
70 nm ITO	102.04	89.45	87.66	99.20

Significant values are in [bold].

**Table 5:** Comparison among our proposed NPWs and other works.

	Ref.^51^	Ref.^52^	Ref.^38^	This work
Periodicity	1000 nm	20 µm	500 nm	400 nm
T_vis_ (%)/R_s_ (Ω/sq)	85.0/15.00	94–97/8–20	88.0/17.2	86.2/8.18
FoMs	148	615–750	160	299.73

## Conclusions

In summary, we demonstrated NPWs as superior flexible TCE, for which, the rationale is to lower the plasma frequency of metals according to the effective media theory. Contrary to conventional metallic meshes whose transparency is governed by Bethe’s diffraction theory, NPW with the smaller openings can simultaneously achieve greater transparency, better conductance, and less Moire effect. To optimize the performance of this NPW-based TCE, we systematically scrutinized the influential factors, including the choice of metals (i.e., Ag, Au, and Al) and the geometric dimensions (i.e., linewidth, periodicity, and thickness) of the NPWs. Our optimized Ag-NPW presented an outstanding performance for the three-color pixels of OLED. For example, the measured transmittance/sheet resistance is 88.3%/9.77 Ω/sq, 87.8%/8.18 Ω/sq, and 88.0%/8.18 Ω/sq for red, green, and blue, respectively. The corresponding FoMs are 443.29, 459.46, and 133.78 in simulation and 300.57, 342.83, and 349.13 in measurements, respectively. All these FoMs are much greater than ITO, a fragile TCE material that prevails in modern optoelectronic devices. Based on these superior mechanical, optical, and electrical properties aforementioned, such flexible Ag-NPW TCE can be readily applied for high-resolution televisions, smartphones, VR/AR displays, solar cells, and other optoelectronic applications.

## Methods

### FDTD simulation

Finite difference time domain simulations were conducted using commercial software, Lumerical, to examine the performances of our proposed NPWs. In simulations, a plane wave light source with a wavelength range from 400 to 800 nm was illuminated along the z-axis under normal incidence. Perfect match layers were set at the z-axis and periodic boundaries were applied along both x- and y-axis. The proposed NPW was on a silica substrate with a refractive index of 1.45 and the environment was air; metals including Au, Ag, and Al followed Drude models, i.e., $\varepsilon_{r} = 1 - \frac{{\omega_{p}^{2} }}{{\omega^{2} - j\omega \gamma }}$, where ${\upgamma }$ is the damping frequency and ω_p_ is the plasmon frequency.

### Sample fabrication

To fabricate our proposed NPWs, we conducted e-beam lithography procedures including e-beam lithography, e-beam evaporation system, and liftoff process in a cleanroom. We first clean glass substrates through piranha solution (H_2_SO_4_:H_2_O_2_ = 1:3) for 1 min before sample fabrication. A PMMA (A4) resists approximately 200 nm for electron beam writing was spin-coated on the cleaned glass substrate; the sample was then soft-baked at 180 °C for 3 min, followed by coating a thin layer of conductive polymer Espacer 300Z (Showa Denko) with a baking temperature of 80 °C for 3 min. The PMMA resist was patterned by electron beam lithography system (Elionix, ELS-7800) with an acceleration voltage of 80 kV and a dosage of 400 μC/cm^2^. The sample was then developed in a solution of methyl isobutyl ketone: isopropanol (IPA) = 1:3 for 1 min and IPA for 25 s. A continuous nanonetwork was patterned on the substrate. The patterned substrate was deposited with 3-nm-thick Ti and metal for specific thickness by the electron beam evaporator. Liftoff was finally carried out in acetone solvent under the assistance of an ultrasonic machine.

## Supplementary Information

Supplementary Information.

## Data Availability

All data generated or analyzed during this study are included in this published article (and its Supplementary Information files).
