# Supplementary material for: Nanoscale plasmonic wires with maximal figure of merits as a superior flexible transparent conducting electrode for RGB colors
Source: Sci Rep. 2022 Jun 30;12:11029. doi: 10.1038/s41598-022-14756-z (PMC9246941; doi:10.1038/s41598-022-14756-z)

**Supporting Information**

**Nanoscale plasmonic wires with maximal figure of merits as a superior flexible transparent conducing electrode for RGB colors**

Chin-Chien Chung^1^, Dong-Sheng Su^1^, Tsung-Yu Huang^2^, Cheng-Yi Lee^1^, Robert Jan Visser^3^, B. Leo Kwak^3^, Hyunsung Bang^3^, Chung-Chia Chen^3^, Wan-Yu Lin^3^ & Ta-Jen Yen^1,*^

^1^ Department of Materials Science and Engineering, National Tsing Hua University, Hsinchu, R.O.C., Taiwan 30013

^2^ Department of Materials Engineering, Ming Chi University of Technology, New Taipei, R.O.C., Taiwan 24301

^3^ Advanced Technology Group, Corporate CTO Office, Applied Materials, Santa Clara, California, USA.

To whom correspondence should be addressed. E-mails: [tjyen@mx.nthu.edu.tw](mailto:tjyen@mx.nthu.edu.tw)

**Diffraction from the nanoscale-plasmonic wires.**

When the incident wavelength coincides with the periodicity of the nanoscale-plasmonic wires (NPWs), the first order diffraction mode would be excited. Some part of incident energy will be then scattered to other angles, thus resulting in a decrease of transmittance. To evidence such mechanism, we simulated the NPWs with a periodicity of 700 nm, a width of 100 nm, a thickness of 50 nm and an incident beam of the Gaussian profile. We monitored the filed distributions at two wavelengths of 650 and 750 nm, i.e., one below the periodicity of the NPWs and the other above. From Fig. S1, we found out the scattered light propagating at inclining angles of$\pm$39.2$^{\circ}$ at 650 nm while the planar wavefront could be observed for the one at 750 nm. Thus, one can maximize the transmittance of the NPWs by reducing the periodicity of the NPWs smaller than the working wavelength.


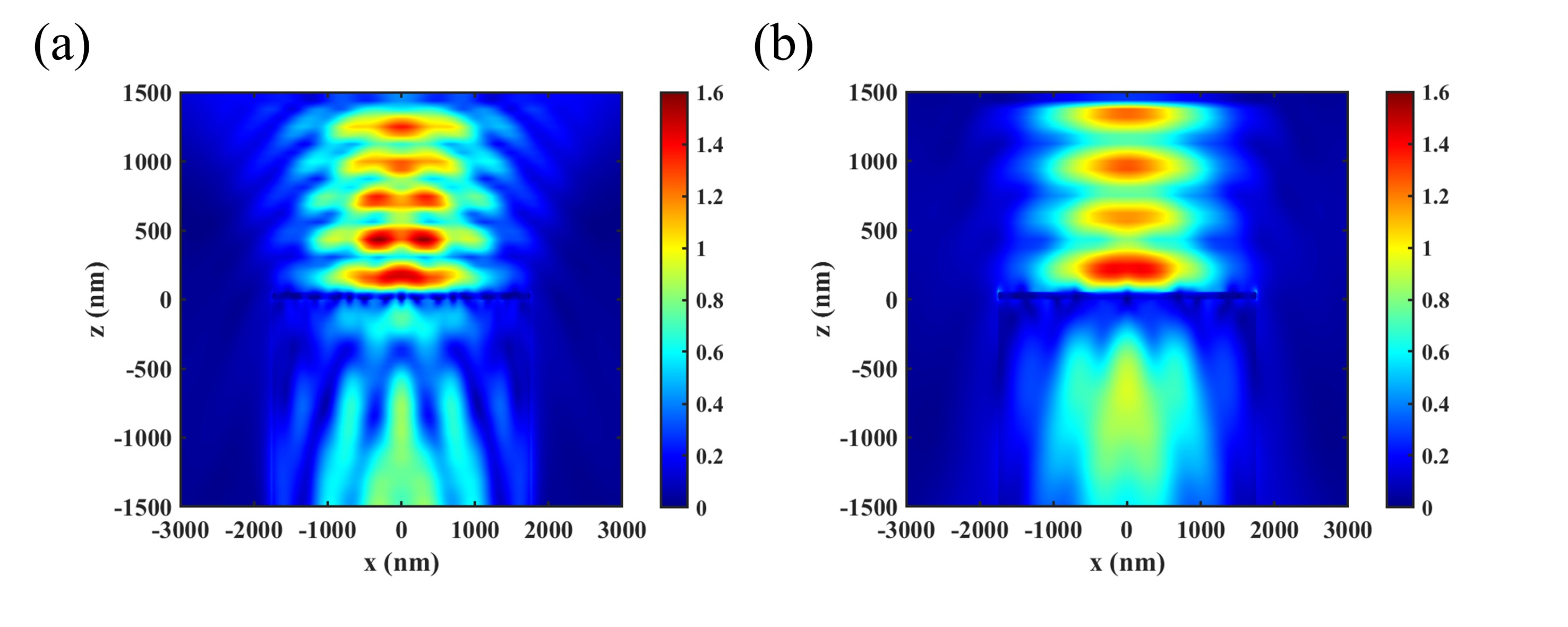


**Fig. S1.** Field Profile of the nanoscale plasmonic wires (NPWs) at a wavelength of 650 and 750 nm. (a) $\pm1$ diffraction modes are excited at a wavelength smaller than the periodicity of NPWs. (b) In comparison, no diffraction mode could be observed at a wavelength greater than the periodicity of NPWs.

**Fig. S2.** Ag NPW fabricated on flexible PET substrate. After bending, the structure of the NPWs under SEM is still robust without any fractures.


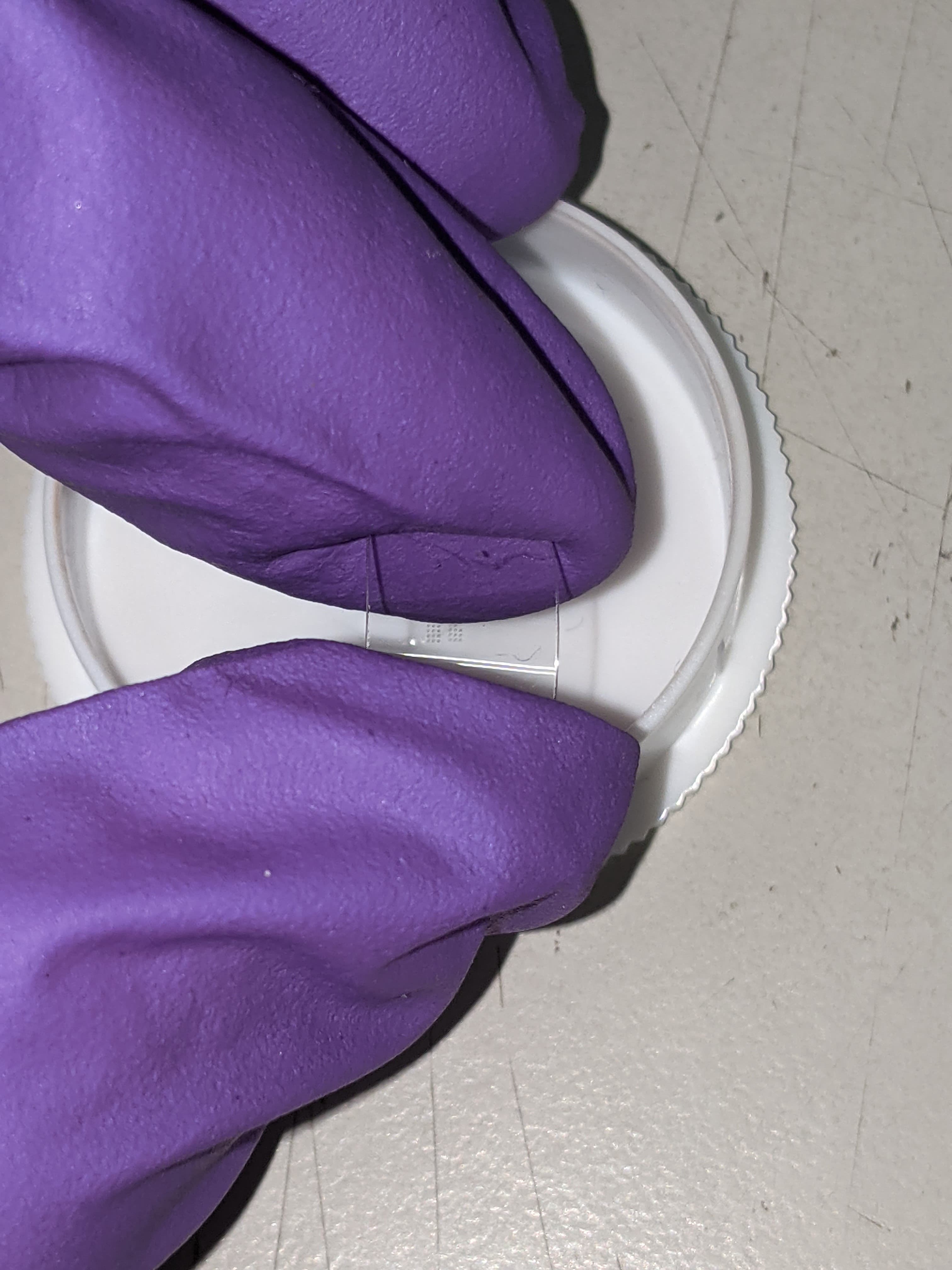

Supplement: Supplementary file 1 — Supplementary Information. [file 41598_2022_14756_MOESM1_ESM.docx]
